# Endogenous Ouabain and Related Genes in the Translation from Hypertension to Renal Diseases

**DOI:** 10.3390/ijms19071948

**Published:** 2018-07-03

**Authors:** Marco Simonini, Paola Casanova, Lorena Citterio, Elisabetta Messaggio, Chiara Lanzani, Paolo Manunta

**Affiliations:** Genomics of Renal Disease and Hypertension Unit, IRCCS San Raffaele Scientific Institute, Università Vita Salute San Raffaele, 20132 Milan, Italy; simonini.marco@hsr.it (M.S.); casanova.paola@hsr.it (P.C.); citterio.lorena@hsr.it (L.C.); messaggio.elisabetta@hsr.it (E.M.); lanzani.chiara@hsr.it (C.L.)

**Keywords:** cardio-tonic steroids, endogenous ouabain, adducin, hypertension, renal damage

## Abstract

The endogenous ouabain (EO) is a steroid hormone secreted by the adrenal gland with cardio-tonic effects. In this article, we have reviewed and summarized the most recent reports about EO, particularly with regard to how it may interact with specific genetic backgrounds. We have focused our attention on the EO’s potential pathogenic role in several diseases, including renal failure, essential hypertension and heart failure. Notably, these reports have demonstrated that EO acts as a pro-hypertrophic and growth-promoting hormone, which might lead to a cardiac remodeling affecting cardiovascular functions and structures. In addition, a possible role of EO in the development of acute kidney injury has been hypothesized. During the last decays, many important improvements permitted a deeper understanding of EO’s metabolisms and functions, including the characteristics of its receptor and the effects of its activation. Such progresses indicated that EO has significant implications in the pathogenesis of many common diseases. The patho-physiological role of EO in the development of hypertension and other cardiac and renal complications have laid the basis for the development of a new selective compound that could selectively modulate the genetic and molecular mechanisms involved in EO’s action. It is evident that the knowledge of EO has incredibly increased; however, many important areas remain to be further investigated.

## 1. Introduction

The cardiac glycosides are a class of drugs derived from the leaves of the *Digitalis purpurea* with a positive inotropic effect on the heart. For a very long time, they were successfully used as a primary treatment for congestive heart failure and arrhythmias [1]. The mechanism of action of the cardiotonic steroids in the human heart has been widely studied, and it is now accepted that it consists in the inhibition of the Na^+^/K^+^-ATPase—a transmembrane enzyme that regulates the gradient of sodium and potassium across the plasma membrane. It was assessed that the inhibition is made through the binding to a highly conserved extracellular recognition sequence of the Na^+^/K^+^ pump [2]. The favorable result of the use of the plant-derived cardiotonic steroids led to hypothesizing the existence of an endogenous cardiac glycoside counterpart in mammals and to assuming that its functional receptor might be the Na^+^/K^+^-ATPase [3]. This enzyme consists of an α- and a β-subunits. The α-subunit is made of 10 transmembrane segments that include, on the extracellular loops, the binding region for the cardiac glycoside, known as the ouabain-binding site. This region is highly conserved in the evolution among species from drosophila to rodents, sheep and humans [2,4].

## 2. Endogenous Ouabain (EO) and Na^+^/K^+^-ATPase Interaction

The hypothesis of the existence of an endogenous cardiac glycoside became solid, when several animals [5,6,7,8,9,10] and human [11,12] studies showed that, in the context of a volume-expanded condition, it was possible to find an endogenous humoral substance that could counterbalance the increased renal reabsorption of sodium and water by the inhibition of Na^+^ transport through vascular and tubular cell membranes. The volume expansion might be a stimulus for the release of this substance, called natriuretic hormone, which could control sodium homeostasis through the inhibition of the key enzyme in the process of its tubular reabsorption, the Na^+^/K^+^-ATPase [13,14]. The effect of the endogenous cardiac glycoside is not limited to the kidney, but also involves the Na^+^/K^+^ pumps in other regions, such as the neuro-vascular system. The consequence of this enzyme’s inhibition is the increase of the intracellular sodium concentration, which is exchanged for calcium through the Na^+^/Ca^+^ exchanger, particularly active in cardiac mussels and smooth vascular muscles [15]. The increase of the intracellular concentration of these two ions, promoted by elevated levels of this endogenous inhibitor, might augment vascular tone determining peripheral vasoconstriction and might lead to a rise in blood pressure [16,17]. Notably, increased levels of cardiotonic glycosides were found in low renin (i.e., volume expanded) hypertension [18]. The vasopressor effect of the cardiotonic steroids has acute and chronic aspects. The acute pressor effect is mediated by the increase in the calcium concentration that causes vasoconstriction, when the short-term cardio-vascular reflexes are blocked. In the case of sustained and chronic elevation of circulating cardiotonic steroids, the pressor effect is maintained by the activation of a signaling pathway that up-regulates the expression of several ion transports in arterial myocytes [19]. It was initially hard to demonstrate that an endogenous digitalis actually exists, and during past years, many research groups have tried to identify it, particularly under physiological and pathological conditions, such as hypertension, pregnancy and neonatal period. Consistent findings obtained over the years suggested that it was possible to isolate different candidate inhibitors of the Na^+^-pump in mammalian tissues, urine and plasma, and several natriuretic substances able to inhibit sodium pumps were identified [4]. Among this compound, it was isolated and characterized the one presenting the majority of the functional properties of the plant-derived cardiac glycoside. Hamlyn’s and Haupert’s groups were the first to describe the presence of a cardiotonic steroid indistinguishable from ouabain in human plasma in 1991 [20]. The endogenous ouabain (EO) was then isolated from bovine adrenal glands [21], human adrenal glands [22], bovine hypothalamus [23], rat adrenomedullary cells [24] and biological fluids [25,26] by using the high performance liquid chromatography (HPLC) and immunoassay methods. The presence of EO was finally demonstrated with mass spectrometry, nuclear magnetic resonance (NMR) and chromatography, confirming its existence and its identicalness to the plant-derived ouabain [27,28]. Furthermore, it was possible to identify the most important production site of EO in the adrenal cortex [29,30]. All this evidence led to the identification in mammals of numerous endogenous cardiotonic steroids as cardenolides and bufodienolides (as marinobufagenine) [31]. However, these endogenous compounds are different from each other and it is fair to assume that they could play a distinct patho-physiological role, acting as tissue-specific regulators of different isoforms of the Na^+^/K^+^-pump [19].

## 3. Endogenous Ouabain Pressor Mechanism and Genes Involved in the Pathogenesis of Hypertension

**Figure 1:**
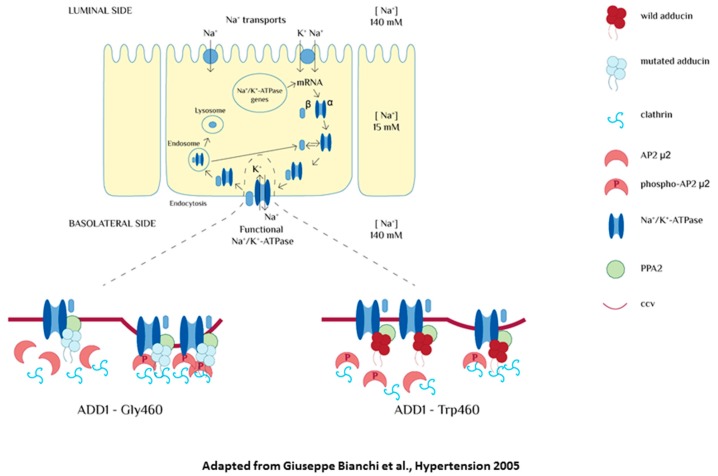
Effect of α-adducin of Na^+^/K^+^-ATPase pump. The mutated form of α-adducin reduces endocytosis, leading to an over-expression of the Na^+^/K^+^ pump molecules on the basolateral membrane and to an increased sodium reabsorption. In the basal condition, the association between phosphatase A2 (PPA2) and adducin is reduced in tubular cells transfected with mutated adducin. The impairment of this cycle may represent the molecular mechanism underlying the reduced endocytosis observed in the presence of mutated adducin (figure adapted from Bianchi et al., Hypertnsion 2005) [3].

During the past years, many research groups investigated the molecular basis of essential hypertension, focusing their attention on renal, endocrine, nervous and humoral dysfunction. In particular, they hypothesized that alterations in renal sodium management could have a key role in its pathogenesis. The Na^+^/K^+^-ATPase activity in the kidneys is regulated by hormonal and genetic factors including EO and the gene coding for α-adducin (*ADD1*). Adducin is a cytoskeletal protein consisting of two heterodimers (α/β or α/γ). *ADD1*, *ADD2* and *ADD3* are the three coding genes for these subunits. It was shown that a polymorphism in the gene *ADD1* (determining the presence of a tryptophan instead of a glycine in the amino-acid position 460, Gly460Trp) was associated with a higher expression of Na^+^/K^+^-ATPase in the surface of the cell and an enhancement of its activity. To better comprehend the mechanisms undergoing primary hypertension, researchers developed several rat models of genetic hypertension, including the Milan hypertensive strain (MHS) of rats that represents a suitable model for a subgroup of human patients with hypertension. It was shown, in both MHS rats and humans, that increased concentrations of EO corresponded to an increased tubular sodium reabsorption and, consequently, hypertension [32]. Furthermore, a prolonged infusion of low doses of plant-derived ouabain in normotensive rats and rat renal tubular cultured cells was associated to an enhanced expression and activity of the Na^+^/K^+^ pump, leading to a reversible form of hypertension. Starting from these evidences, it was hypothesized that ouabain itself may be considered as a pressor agent in vivo. The same phenomenon was documented in cells transfected with genetic variants of the MHS adducin [33]. In the following years, researchers also tried to understand the mechanism, by which both ouabain and mutated adducin could modify the expression of the Na^+^/K^+^-ATPase in the kidneys. Under these two conditions, it was possible to evidence a slower recycling of the sodium pump from the surface of the cell and, consequently, an excessive expression of the Na^+^/K^+^-ATPase in light of a tighter anchoring to the cytoskeletal proteins. This is the biochemical alteration present in both the ouabain- and adducin-dependent forms of hypertension [34]. These findings apparently contradict the traditional natriuretic hypothesis that considers the cardiotonic steroids as inhibitors of the Na^+^/K^+^-ATPase. According to this hypothesis, volume expansion conditions might induce the release of an endogenous hormone (EH) able to promote natriuresis. High levels of EO should lead to a decrease, rather than an increase, of Na^+^/K^+^-ATPase activity. To clarify this issue, the relation between EO and changes in sodium balance was studied in both rats and patients with essential hypertension. The results showed that an acute and chronic restriction of salt intake (but not the acute salt loading) was associated with a significant rise in EO plasmatic levels [35]. Consequently, conditions of salt and water reductions might provoke the elevation of the EO humoral concentration, meaning that EO does not act as a natriuretic hormone in vivo [4,36,37]. An important augmented EO level during physical exercise was demonstrated, which is a state characterized by an increased sympathetic activity and a decline in renal blood flow [4,38], and is found in patients undergoing cardiac surgery [39]. These results evidenced that EO induces a variety of important mechanisms, which augment vascular tone promoting renal sodium retention [4]. It might be a fair assumption that EO, through the enhancement of the renal Na^+^/K^+^-ATPase activity, plays a role in body sodium homeostasis and in the re-establishment and maintenance of the hydro-saline equilibrium [3,19]. Previous studies demonstrated that adducin has a direct role in the modulation of the renal Na^+^/K^+^-ATPase (Figure 1). Following this evidence, it was important to understand whether adducin polymorphism (Gly460Trp) might influence EO’s response of the adaptation to a low-salt diet.

**Figure 2:**
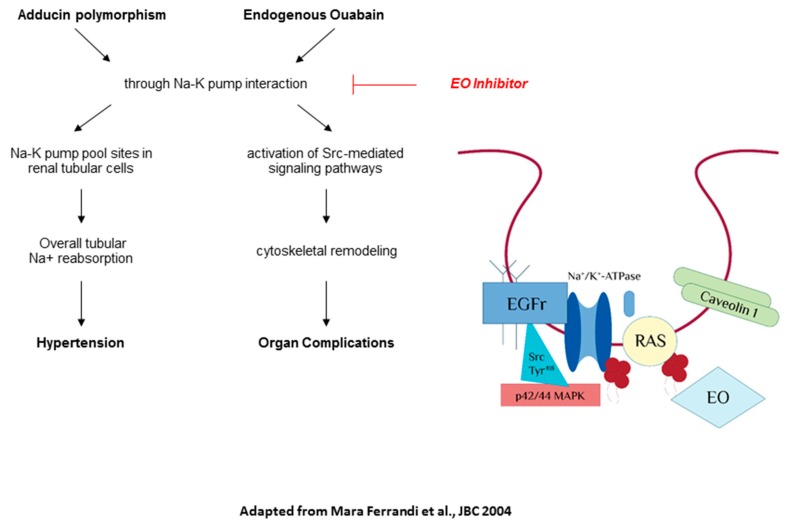
Physio-pathological interaction between Endogenous Ouabain (EO) and of α-adducin. This interaction can lead to the development of hypertension and organ maladaptive remodeling and potential target of an anti-ouabain compound (as rostafuroxin) (figure adapted from Ferrandi et al., JBC 2004) [43].

In hypertensive patients with mutated *ADD1* gene (*Trp-460*), a chronic low-salt diet is associated to an important augment of EO plasmatic concentration. Contrarily, it is not possible to recognize the same condition in wild-type patients (Gly-460), thus counteracting the hypotensive effect of the low-salt diet. Similarly, there is an increase of EO levels in MHS rats and the congenic rat strain NA (obtained by the introgression of the MHS *ADD1* locus into the normotensive genetic background), but not in normotensive rats. A high-salt diet does not modify plasma EO in rats, nor in humans. These data suggest that adducin genotype might predict the changes of EO plasmatic levels under salt restriction conditions [40,41]. Another study examined the linkage between EO and blood pressure in the general population obtaining several new evidences. Notably: (1) people with the Gly460Trp polymorphism of α-adducin have higher EO plasmatic levels compared to the carriers of the wild-type genotype; (2) the EO plasmatic concentration is directly proportional to urinary potassium excretion; and (3) there is an important interaction between blood pressure, EO levels and urinary sodium excretion [42]. We can finally affirm that EO acts as a positive regulator of blood pressure during chronic low-salt diet, whilst it prevents high salt-induced blood pressure when the salt intake is elevated. There is a correlation between EO and genetics in the homeostatic regulation of blood pressure in response to changes in salt intake (Figure 2). However, this complex relationship requires further investigation in order to be fully clarified.

## 4. The Correlation between Endogenous Ouabain and Organ Damage

### 4.1. Endogenous Ouabain and Cardiovascular Disease

Studies on rat (normotensive, MNS and MHS rats) and human models have demonstrated that EO contributes not only to the pathogenesis of hypertension, but also to the development of cardiac complications, such as ventricular hypertrophy, heart failure and acute myocardial infarction [12,13,14]. Approximately half of patients affected by essential hypertension have high circulating levels of EO, and previous studies demonstrated a direct relation between increased EO levels, left ventricular mass indices and stroke volumes, whilst a negative correlation of increased EO levels with heart rates was seen [15,16,17]. EO might be actually considered as a growth-promoting hormone. Indeed, many authors showed that EO is involved in pro-hypertrophic and pro-fibrotic pathways that lead to cardiac remodeling with a negative impact on both cardiac structures and cardiovascular functions [30,44]. The authors also tried to understand how EO might perform the role of a signal transducer. Studies on cultured vascular cells showed that EO, after the binding to its receptor (Na^+^/K^+^-ATPase), could trigger a tyrosine-kinase protein starting an intracellular-signaling cascade that constitutes a stimulus for the epidermal growth factor receptor (EGFR). This pathway finally permits the transcription of gene encoding for pro-fibrotic factors [45], promoting cardiac hypertrophy. Relevant evidences showed that a chronic activation of this complex protein-kinase cascade could finally lead to heart failure [46]. It was hypothesized that the molecular pathway that leads to organ hypertrophy in vivo was similar to the one described in cultured vascular cells [16]. In a study conducted on more than 800 patients undergoing elective cardiac surgery, it was supposed that EO might be also used as a valuable biomarker of heart failure. This study confirmed the already hypothesized negative relation that intercourses between increased EO levels and left ventricular ejection fraction and the positive correlation between EO levels and cardiac end-diastolic diameters. EO were dosed in all of these patients after and before the surgery, and it was seen that higher EO circulating levels both in the pre-operation and the immediate post-operation were associated with worst cardiovascular presentation, higher morbidity and increased risks of perioperative mortality after cardiac surgery [47].

### 4.2. Endogenous Ouabain and Renal Disease

The main actor in cardiac glucosides’ metabolism is the liver; however, it is now clear that kidneys also have an important role in their clearance. To validate this evidence, experiments conducted on the rat demonstrated that partial nephrectomy was associated with higher EO circulating levels [12]. Similarly, it was seen that the progression of kidney disease, in particular the end-stage renal failure, was associated with an increase of EO levels [19]. High concentrations of EO are comparable to an excessive and not controllable digitalization that might lead to a vasopressor effect and other important cardiac side effects. Indeed, studies on patients with Chronic Kidney Disease (CKD) or undergoing dialysis demonstrated that elevated levels of EO were strongly associated with alterations in ventricular mass and geometry [48,49], independently from blood pressure and other determinants of left ventricular hypertrophy. The comorbidities that characterize patients with severe renal diseases and dialyzed patients, such as hypertension and cardiac hypertrophy, might thus be potentially related to EO. These data stimulated the interest of the impact of EO on the renal function. To understand this aspect, a selective podocyte marker protein called nephrin was used. Studies on rats showed that a prolonged exposure to high levels of EO was associated with a less expression of nephrin in the podocyte, a reduction in creatinine clearance and increased proteinuria. This last finding was also replicated ex vivo with the incubation of low dose of ouabain in podocyte primary cell cultures [50]. EO modulates the Na^+^/K^+^-ATPase that is involved in tubular ischemic damage, and it is responsible for the initial nephrinuria and the glomerular damage. The EO’s promotion of kidneys’ damage shown in rat models suggested a possible role of EO in acute kidney injury (AKI). In a recent observational study, a blood sample for the dosage of EO was taken during the induction of anesthesia in patients destined to elective cardiac surgery. Interestingly, patients with higher preoperative levels of EO were characterized by worst renal outcomes and higher mortality rates [47,50,51]. Starting from these evidences, it is a fair assumption that EO might be considered to be a valuable biomarker of individual susceptibility to the development of AKI after cardiac surgery [52,53].

Recent studies individuated an additional effect of EO, showing that it might also act as a pro-cystogenic agent in the development of autosomal dominant polycystic kidney disease (ADPKD). It was shown that the Na^+^/K^+^-ATPase of ADPKD cells has an increased affinity with EO, and even if the mechanism undergoing this abnormal affinity remains uncertain, it might enhance ADPKD cell’s susceptibility to circulating EO. The exposure of primary cultures of cells isolated from renal cysts of ADPKD patients to nanomolar concentrations of EO provokes the proliferation of cyst epithelial cells. In contrast, similar concentrations of EO had a pour influence on the proliferation of normal human kidney cells [52,53].

### 4.3. Endogenous Ouabain and Brain Disease

The presence of EO inside the central nervous system (CNS) is well-known since the early 1990s [54,55]. It was demonstrated that it is an integrated component of a hypothalamic renin-angiotensin-aldosterone system (RAAS) and also plays an important role in regulation of systemic Blood Pressure (BP) [37,40,56]. The relation between the central and peripheral RAASes with central and peripheral EO is still not completely understood [55], but it was discovered that EO-induced signaling in neurons had positive and direct consequences on rat brain in terms of brain cells survival [57]. Similarly, in mouse models, the reduction of endogenous steroids seems to have a protective effect on oxidative stress for CNS [58]. Endogenous steroids, in particular EO-like compounds, were also described as potential risk factors involved in the etiology of bipolar disorder [59], mania [60] and depression [61]. Moreover, the use of anti-ouabain antibodies showed a reduction of maniac [60], as well as depressive [62] status. The development of EO associated behavior disorders seems directly associated with a dys-regulation of Na^+^/K^+^-ATPase inside CNSes [59,60].

### 4.4. Endogenous Ouabain as Therapeutic Target

As already said in this review, several studies during the last decays individuated a central core in the increased EO levels that could connect the pathogenesis of several cardiac and renal diseases, including hypertension, cardiac hypertrophy, heart failure, renal failure and ADPKD. This fundamental evidence stimulated the birth of a new research branch aiming to individuate a selective competitor of EO, paving the way for the formulation of new antihypertensive agents that could selectively correct the molecular mechanisms behind. On this basis, authors’ research groups developed a new digitoxigening-derived compound, called Rostafuroxin. Its mechanism of action expects to display EO from its specific binding sites on the Na^+^/K^+^-ATPase, modulating its abnormal expression in the cell surface without inhibiting other renal sodium transporters and without influencing other hormonal pathways [19,43,63]. As already mentioned, the interaction between EO and its receptor triggers a complex cascade of intracellular second-messengers that ends with the generation of hypertrophic stimuli. It was seen that nanomolar concentrations of Rostafuroxin could antagonize the interaction between EO and the Na^+^/K^+^ pump, normalizing this signal response and blocking the excessive activation of EGFR [19]. In both normotensive and MHS rat models, low oral doses of Rostafuroxin could normalize the up-regulation of renal Na^+^-pump, leading to a reduction of blood pressure levels [19,63]. A similar effect was obtained with the use of Rostafuroxin in other rat models, such as the deoxycorticosterone acetate–salt rat and the reduced-renal-mass hypertensive rat. Both models were characterized by a condition of low plasmatic renin, volume expansion and increased levels of EO [64]. In contrast, it was interesting to evaluate that Rostafuroxin does not act as an antihypertensive agent in these models, in which EO and α-adducin polymorphism are not implicated in the pathogenesis of hypertension, such as the normotensive control rats and spontaneous hypertensive rats. Moreover, preliminary results suggest that this molecule, used at oral doses of 7–10 μg/kg/day, might revert the ouabain-induced hypertrophic activity [35,37]. In a trial of unselected patients, it was shown that Rostafuroxin does not influence blood pressure in these patients with no elevated EO levels, assuming that its effect on blood pressure is strictly related to the genetic background that regulates the synthesis and the clearance of EO. It was assessed that in Rostafuroxin-sensitive patients, there is a decline of systolic blood pressure of 14 mmHg after 4 weeks of treatment [65]. Nowadays, we do not have data about the effect of Rostafuroxin on the treatment of renal failure and the effects of hypertension on patients with end-stage renal disease, but this compound might have the effect of amelioration of the grade of hypertrophy and heart failure [46]. The pharmacological profile and the selective mechanism of action of Rostafuroxin make it the prototype of a compound devoid of the cardiovascular and hormonal side effects associated with digitalis and diuretic. Indeed, there was no evidence of intrinsic cardiac inotropic effects or arrhythmogenic activity.

## 5. Conclusions

During the last years, numerous important advances have led to a better understanding of EO’s metabolisms and functions, including the characteristics of its receptor and the molecular effects of its activation. Although many important areas need to be further investigated, compelling data reinforce the concept that high EO plasmatic levels and adducin polymorphism (Gly460Trp) are associated with an increased risk of developing diseases including hypertension, ADPKD and organ complications, such as podocyte injury, cardiac and kidney hypertrophy. It also contributes to the development and the maintenance of AKI in critically ill patients.

The importance of these evidences is that the understanding of the patho-physiological mechanism undergoing complex diseases represents a valid substrate to individuate specific pharmacological targets. This is mainly important in complex multifactorial disease, when a tailored approach based on individuals’ phenotypes and genotypes should be chosen to treat individual patients carrying a specific genetic background. Rostafuroxin might be considered to be a revolutionary therapy for hypertension when increased EO circulating levels and adducin polymorphism exert a pathogenetic role. Rostafuroxin perfectly fits the new concept of personalized medicine, and it can be considered to be a safe drug without the most common side effects of previous used compounds.
